# Endoscopic findings of gallbladder lesions evaluated with image‐enhanced endoscopy: A preliminary study using resected gallbladders

**DOI:** 10.1002/deo2.70136

**Published:** 2025-05-03

**Authors:** Kiyoyuki Kobayashi, Maki Ayaki, Takako Nomura, Kaho Nakatani, Masaki Tokumo, Yasutaka Kokudo, Toshiaki Morito, Ichiro Ishikawa, Akihiro Kondo, Yasuhisa Ando, Hironobu Suto, Minoru Oshima, Ryota Nakabayashi, Toshiaki Kono, Naoki Fujita, Hiroki Yamana, Hideki Kamada, Masafumi Ono, Keiichi Okano, Hideki Kobara

**Affiliations:** ^1^ Division of Innovative Medicine for Hepatobiliary and Pancreatology Faculty of Medicine Kagawa University Kagawa Japan; ^2^ Department of Internal Medicine Kagawa Rosai Hospital Kagawa Japan; ^3^ Department of Gastroenterology and Hepatology HITO Medical Center Ehime Japan; ^4^ Department of Gastroenterology and Neurology Faculty of Medicine Kagawa University Kagawa Japan; ^5^ Department of Hepato‐Biliary‐Pancreatic Surgery Kagawa Rosai Hospital Kagawa Japan; ^6^ Department of Pathology Kagawa Rosai Hospital Kagawa Japan; ^7^ Department of Neuropsychiatry Faculty of Medicine Kagawa University Kagawa Japan; ^8^ Department of Gastroenterological Surgery Faculty of Medicine Kagawa University Kagawa Japan

**Keywords:** cholecystectomy, diagnosis, endoscopy, gallbladder, image‐enhanced endoscopy

## Abstract

The diagnosis of gallbladder (GB) lesions relies on imaging findings. Transpapillary cholangioscopy can potentially be used to diagnose GB lesions; however, the images obtained remain unclear. This study aimed to characterize the endoscopic findings of GB lesions. We examined the endoscopic features of GB lesions in 50 consecutive patients who underwent cholecystectomy. GB specimens were obtained immediately following cholecystectomy, opened on the side opposite the liver bed, and flushed with saline solution. Each lesion was assessed using a high‐resolution endoscope equipped with white light and narrow‐band imaging magnification. For elevated lesions, both the surface structure (classified as regular, irregular, or absent) and vascular structure (dilation, meandering, caliber change, non‐uniformity, and loose vessel areas) were assessed. Twelve of the 50 patients had elevated lesions, including cholesterol polyp (*n* = 4), hyperplastic polyp (*n* = 1), xanthogranulomatous cholecystitis (*n* = 1), and GB carcinoma (*n* = 6). Advanced GB carcinoma, as opposed to T1 GB carcinoma, demonstrated a papillary surface with destructive areas and neovascularization on narrow‐band imaging magnification. Endoscopic images of each GB lesion were characterized, and the differences between GB carcinomas and benign lesions were identified. This preliminary classification may contribute to innovative imaging diagnosis and targeted biopsy for diagnosing GB lesions under direct vision.

## INTRODUCTION

The diagnosis of gallbladder (GB) lesions traditionally relies on imaging findings; however, a definitive diagnosis is often challenging. Bile cytology and bile duct biopsy have been used for pathological diagnosis of GB, although the correct diagnosis rate remains low, necessitating improvements in diagnostic performance.^1^, ^2^

For bile duct lesions, the classification of biliary tract lesions as benign or malignant based on cholangioscopic findings and direct visual biopsy using cholangioscopy has been reported,^3^, ^4^ with a marked improvement in diagnostic ability.^5^, ^6^, ^7^ Furthermore, cholangioscopy is expected to enhance the ability to diagnose GB lesions. We have previously reported observations inside the GB and direct visual biopsy findings using a cholangioscope inserted transpapillary into the GB.^8^, ^9^

Image‐enhanced endoscopy (IEE), such as narrow‐band imaging (NBI), has been used for diagnosis by highlighting blood vessels in the gastrointestinal region.^10^, ^11^ The usefulness of IEE for cholangiocarcinoma has also been reported, and further development is anticipated.^12^, ^13^, ^14^ If further developed, the availability of IEE for GB lesions would improve the diagnosis of complex cases.

This study aimed to observe various GB lesions using white light imaging (WLI) and NBI, focusing on the characteristic images of GB carcinoma (GBC). Herein, we present the endoscopic findings of the GB lumen following cholecystectomy.

## PROCEDURE OR TECHNIQUE

### Procedure

GB specimens were obtained immediately following cholecystectomy, opened on the side opposite the liver bed, and flushed with saline solution. Whenever feasible, the GB lumen was examined using high‐resolution endoscopes (GIF‐H290Z or XZ1200; Olympus) while immersed in saline solution.

We captured endoscopic images from areas with macroscopic findings, such as elevation or thickening. NBI observations were performed following conventional WLI. If no macroscopic findings were observed, endoscopic images were obtained from the center of the open specimen.

### Patients and data collection

This prospective observational study was conducted at two facilities between November 2020 and July 2022. Consecutive patients who underwent cholecystectomies during the study period were included.

Written informed consent for this study was obtained from all patients before they underwent cholecystectomy. This study was approved by the Institutional Ethics Review Board of the two participating facilities (Ethics Committee Approval Nos. – Kagawa Rosai Hospital: R2‐22, HITO Medical Center: 20210507002) and performed in accordance with the Declaration of Helsinki.

### Assessment of endoscopic findings

All endoscopic images obtained from each patient were reviewed by two expert endoscopists (Maki Ayaki and Takako Nomura) who had made more than 200 IEE diagnoses for gastrointestinal neoplasms. One of the authors (Kiyoyuki Kobayashi) instructed the evaluators with representative endoscopic images that demonstrated the surface and vascular structures prior to blinded assessment. Interobserver agreement was evaluated between these two endoscopists. The evaluators were blinded to the clinical details of all patients and histological results of the lesions. The final endoscopic diagnosis was confirmed by these two authors based on the histological diagnosis.

For elevated lesions, both the surface structure (classified as regular, irregular, or absent) and vascular structure (dilation, meandering, caliber change, nonuniformity, and loose vessel areas) were assessed. In this study, a regular surface pattern was characterized by glandular structures that exhibited uniform shapes, symmetrical distributions, and consistent arrangements. Irregular surface patterns were characterized by diverse shapes, asymmetrical distributions, and irregular arrangements. The cases where no discernible surface pattern was observed were categorized as those with an absent surface pattern. Vascular dilation was defined as vessels with larger diameters compared to normal GB vessels, caliber variation was defined as changes in diameter within a single vessel, and non‐uniform vascularity was defined as differences in vascular morphology between adjacent regions. In addition to the five vascular characteristics (dilation, meandering, caliber change, non‐uniformity, and loose vessel areas) evaluated in this study, combinations of these findings are defined as neovascularization and destructive vessels. Neovascularization was defined as the formation of new blood vessels associated with tumor growth and characterized by meandering, caliber change, and non‐uniformity, presenting as an irregular, densely branched vascular network. Destructive vessels typically display caliber change, non‐uniformity, and loose vessel areas, with distinctive features of vascular discontinuity and abrupt termination (Figure S1).

After the histological diagnosis was confirmed, the elevated lesions were classified into benign and malignant groups. The surface and vascular structures were compared between the two groups, and statistical tests were conducted.

### Assessment of histological findings

Histological evaluations were performed by pathologists at each facility, focusing on the areas observed during endoscopy according to standard clinical procedures. Cancer diagnosis was based on the 8th edition of the “TNM Classification of Malignant Tumors” established by the Union for International Cancer Control. Furthermore, GBC with histological depth limited to the mucosa or muscularis propria was defined as early‐stage (T1a, T1b), while T2 or higher as advanced‐stage.

### Statistical analysis

Continuous variables are expressed as median (range) or *n*. Categorical variables are expressed as percentages. Fisher's exact test was used to compare the proportions between two independent groups. All *p*‐values were two‐sided, and *p*‐values of ≤0.05 were considered statistically significant. Interobserver agreement was assessed using the kappa (κ) coefficient, which was calculated as κ = (Po‐Pe) / (1‐Pe), where Po represents the observed proportion of agreement and Pe represents the proportion of agreement expected by chance. The strength of agreement was interpreted according to the following criteria: κ >0.8, almost perfect agreement; 0.6–0.8, substantial agreement; 0.4–0.6, moderate agreement; 0.2–0.4, fair agreement; and <0.2, slight agreement. A κ‐value of 0 indicated agreement no better than chance, while negative values suggested systematic disagreement.^15^ Data were analyzed using EZR version 1.55 (https://www.jichi.ac.jp/saitama‐sct/SaitamaHP.files/statmed.html).^16^

## RESULTS

### Patient characteristics

In this study, we prospectively enrolled 50 patients (median age, 67 years; age range, 38–90 years; 24 males, 26 females), of whom six had normal GBs excised during gastric cancer surgery (Table S1). The pathological findings for the remaining specimens are as follows: 26 cases of chronic cholecystitis, two of cholesterosis, four of cholesterol polyps, one of hyperplastic polyps, four of adenomyomatosis, and one of xanthogranulomatous cholecystitis (XGC). GBC was diagnosed pathologically in six patients, with one case classified as pT1a, one case as T1b, two cases as T2, and two cases as T3.

### Endoscopic images

#### Benign lesions

**FIGURE 1:**
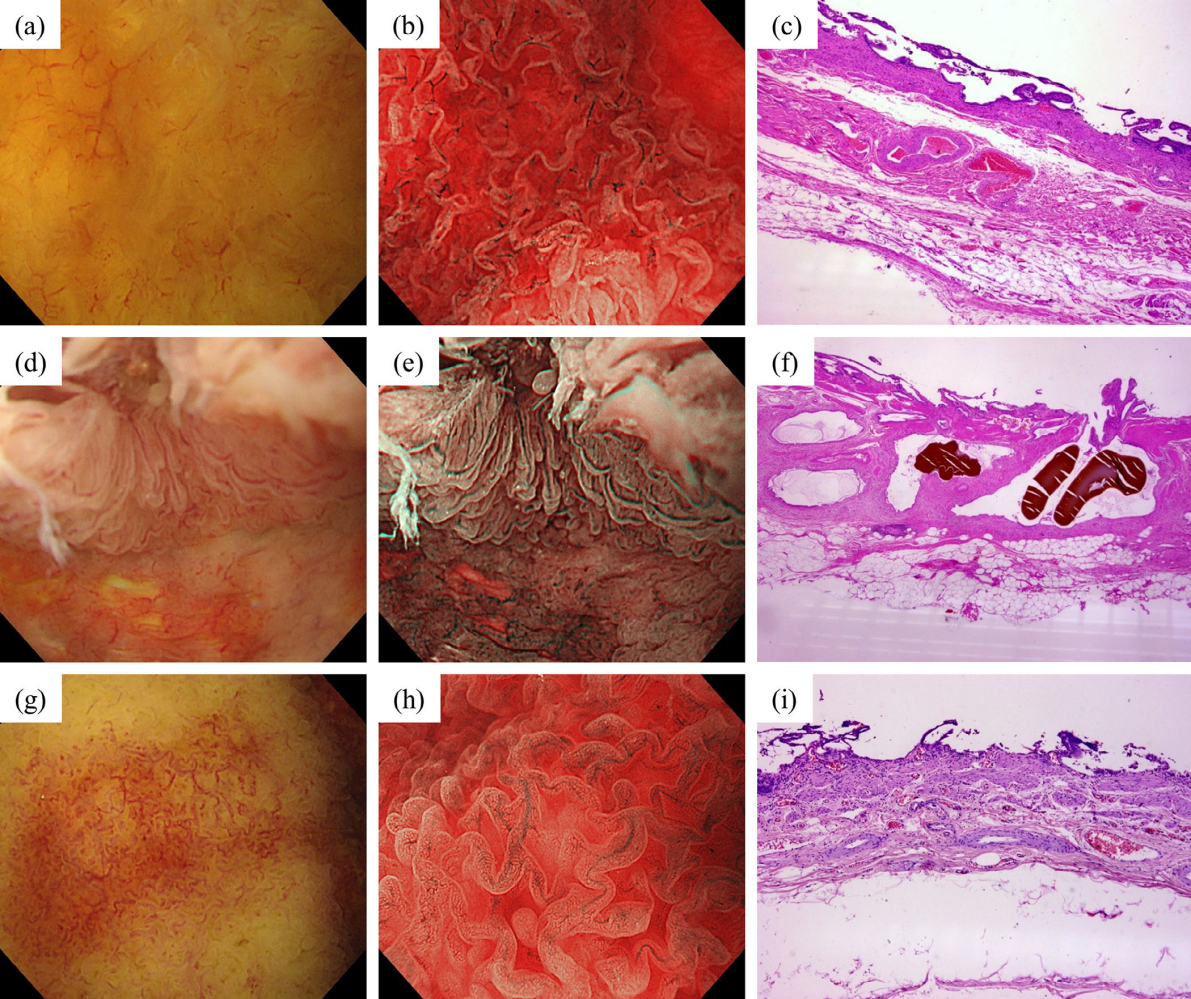
Endoscopic findings of benign gallbladder (GB) lesions. Normal GB: (a) Mucosa showing the regular arrangement of a low‐height columnar epithelial surface structure under white light imaging (WLI). (b) Narrow‐band imaging (NBI) magnification showing regular vascularity without dilation. (c) Histopathological examination confirming no specific inflammatory or neoplastic lesions. Adenomyomatosis of the GB: (d) WLI showing a regular arrangement of columnar epithelium similar to that of a normal GB. (e) Under NBI magnification, the epithelium is taller than that of normal GB. (f) Histopathological examination confirming adenomyomatosis characterized by dilated ducts and smooth muscle. Cholecystolithiasis: (g) WLI showing areas of redness due to vasodilation. (h) NBI magnification showing slightly dilated regular vessels. (i) Histopathological examination revealing mucosal thinning, wall fibrosis, and mild inflammation with lymphocyte accumulation.

The mucosa of normal GBs exhibited a white tone and slightly elevated mucosa, with a low‐height columnar epithelium arranged in a regular pattern under WLI (Figure 1a), observed in the NBI‐enhanced images. NBI revealed regular vascularity without dilation (Figure 1b). Histopathological examination confirmed no specific inflammatory or neoplastic lesions (Figure 1c).

Adenomyomatosis was characterized by a higher columnar epithelium than that in the normal GB using WLI (Figure 1d) and NBI (Figure 1e). Histopathological examination confirmed adenomyomatosis characterized by dilated ducts and smooth muscle (Figure 1f).

In cholecystolithiasis, WLI showed a white tone and short columnar epithelium but appeared reddish due to vasodilation (Figure 1g). NBI revealed slightly dilated and regularly arranged blood vessels (Figure 1h). Histopathological examination revealed mucosal thinning, wall fibrosis, and mild inflammation with lymphocyte accumulation (Figure 1i).

**FIGURE 2:**
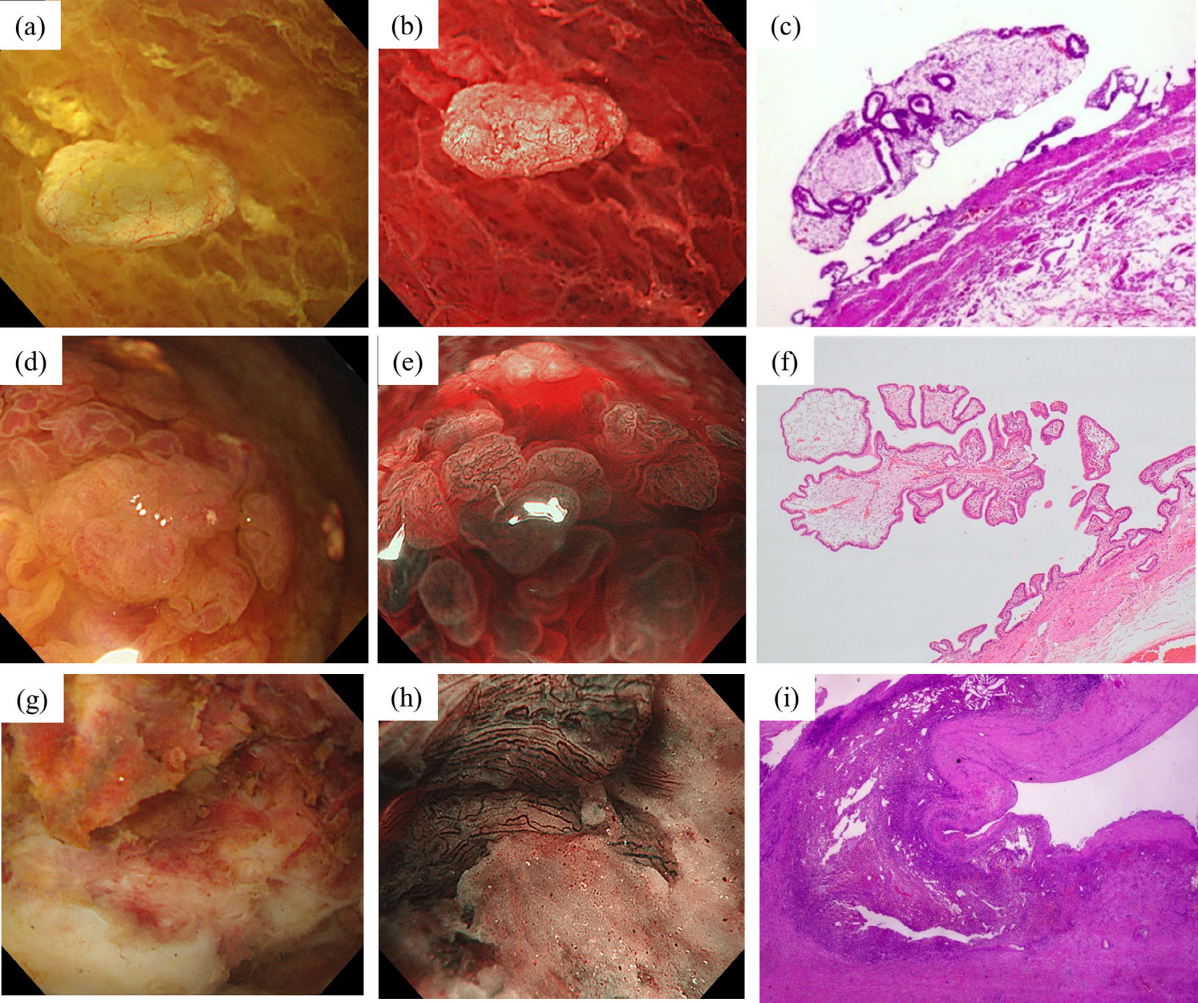
Endoscopic findings of benign elevated lesions. Cholesterol polyp: (a) White light imaging (WLI) showing a yellowish, spherical, elevated lesion with a thin stalk. (b) Narrow‐band imaging (NBI) showing regular vessels without dilation. (c) Histopathological examination showing the lesion covered by non‐dysplastic epithelium, with foam histiocytes infiltrating the interstitium. Hyperplastic polyp: (d) WLI revealing a slightly reddish elevated lesion. (e) NBI magnification showing a regular surface structure but a wide intervening part and slight vascular dilatation. (f) Histopathological examination revealing hyperplastic changes in the ductal epithelium and an increased number of capillaries. Xanthogranulomatous cholecystitis: (g) Under WLI, white and brown tones were noticeable in the ulcer‐scarred area and the nonstructural area where the mucosal epithelium had sloughed off. (h) Under NBI magnification, scarring was observed in areas with an intact surface structure, accompanied by numerous uniformly dilated blood vessels with irregular vascular distributions. (i) Histopathological examination confirming the fibrous thickening of the gallbladder wall, clusters of pigment‐phagocytosing histiocytes, granular inflammatory changes, and abscess formation.

A cholesterol polyp was confirmed as a lesion with a yellowish smooth surface structure and fine blood vessels on WLI (Figure 2a). Under NBI magnification, the surface structure was undisturbed, the vascular structure was regular, and the stromal area appeared whitish (Figure 2b). Histopathological examination showed that the lesion was covered by non‐dysplastic epithelium, with foam histiocytes infiltrating the interstitium (Figure 2c).

A hyperplastic polyp was recognized as a reddish‐elevated lesion with a regular lobulated surface under WLI (Figure 2d). NBI magnification revealed a regular lobulated surface with a wide intervening area and slight vascular dilatation (Figure 2e). Histopathological examination revealed hyperplastic changes in the ductal epithelium and an increased number of capillaries (Figure 2f).

In XGC, white and brown tones in the ulcer‐scarred and nonstructural areas where the mucosal epithelium had sloughed off were under WLI (Figure 2g). Under NBI magnification, scarring was observed in areas where the surface structure was intact, and many uniformly dilated blood vessels with irregular vascular distributions were present (Figure 2h). Histopathological examination confirmed fibrous thickening of the GB wall, clusters of pigment‐phagocytosing histiocytes, granular inflammatory changes, and abscess formation, consistent with xanthogranulomatous inflammation. Furthermore, no obvious neoplastic changes or findings suggestive of malignancy were observed in the sections (Figure 2i).

#### Adenocarcinoma

**FIGURE 3:**
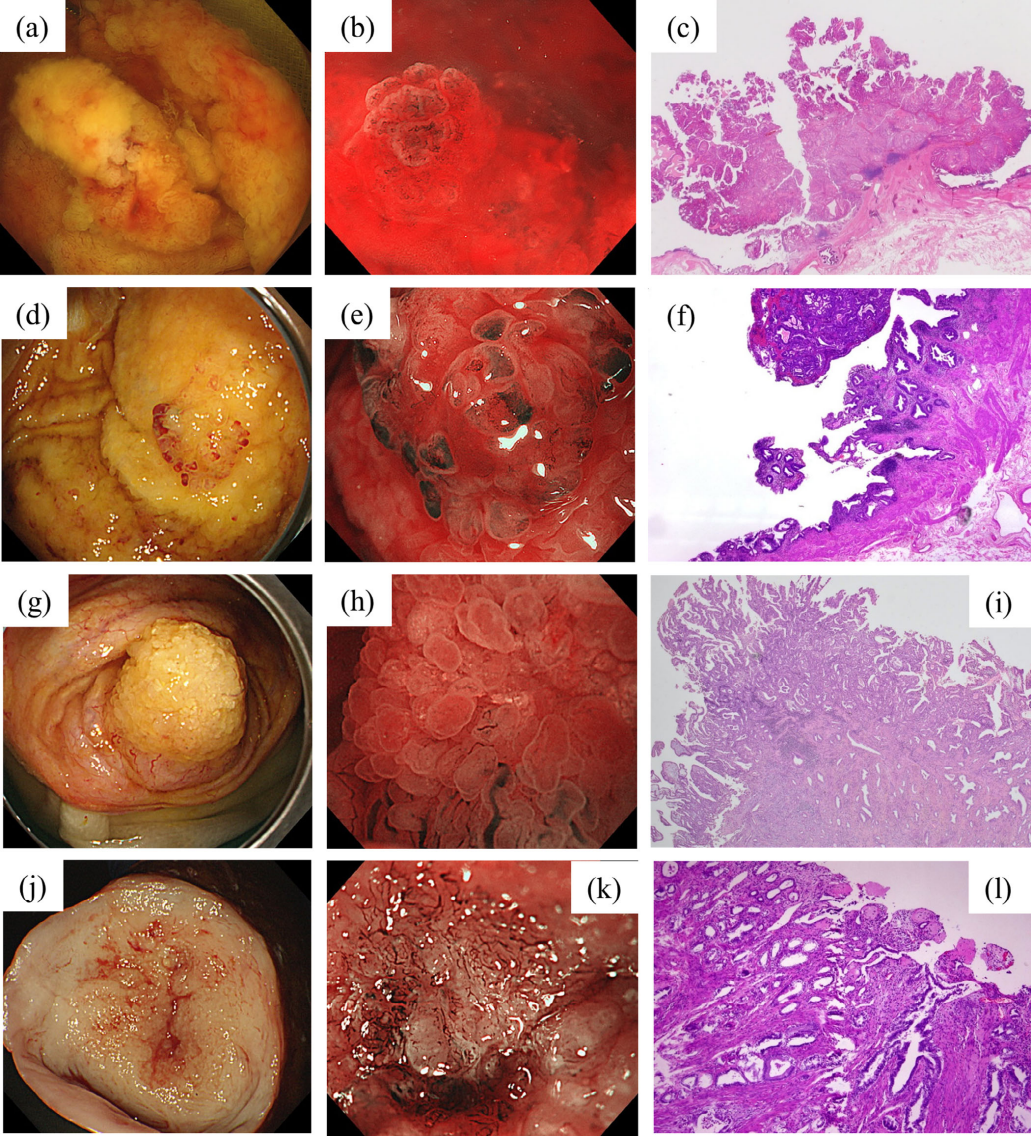
Endoscopic findings of gallbladder carcinomas. T1a gallbladder carcinoma (GBC): (a) White light imaging (WLI) revealing an elevated lesion with a thick stalk. (b) Narrow‐band imaging (NBI) magnification showing that the surface structure was papillary and the vascular structure was caliber changes with an irregular distribution of blood vessels. (c) Histopathological examination revealing atypical cells with tubular proliferation in the pedunculated lesion, with no invasion of the muscularis mucosae observed. T1b GBC: (d) WLI showing a nodular elevated lesion with an irregular papillary surface structure. (e) NBI magnification revealing caliber changes with an irregular distribution of vessels, which were slightly more dilated than those observed in T1a GBC. (f) Histopathological examination revealing that the adenocarcinoma had infiltrated the muscularis propria of the GB but showed no subserosal invasion. T2 GBC: (g) WLI showing a large elevated lesion with a papillary surface structure, although some areas were non‐structured. (h) NBI magnification revealing neovascularization and greater vascular disparity than T1 GBCs. (i) Histopathological examination revealing an adenocarcinoma with small‐ to medium‐sized tubular infiltration extending into the subserosa. T3 GBC: (j) WLI showing a large, partially submucosal, tumor‐like nodule. (k) NBI magnification revealing irregular vessel images with neovascularization and destructive vessels. (l) Histopathological examination revealing a tubular adenocarcinoma proliferated invasively into the liver parenchyma.

Early‐stage GBC (T1a) was recognized as an elevated lesion with a thick stalk under WLI (Figure 3a). The magnified image had no unstructured areas in the surface structure, and the surface was papillary, resembling a salmon roe. NBI magnification revealed caliber changes with an irregular distribution of blood vessels (Figure 3b). Histopathological examination revealed atypical cells with tubular proliferation in the pedunculated lesion, identified as a moderately differentiated adenocarcinoma. The carcinoma corresponded to the elevated lesion, with no invasion of the muscularis mucosae observed (Figure 3c).

Early‐stage GBC (T1b) was identified as a nodular elevated lesion with an irregular papillary surface structure under WLI (Figure 3d). NBI magnification revealed caliber changes with an irregular distribution of vessels, which were slightly more dilated than those observed in T1a GBC (Figure 3e). Histopathological examination revealed an adenocarcinoma with a papillary to tubular growth pattern. The tumor cells had infiltrated the muscularis propria of the GB but did not show subserosal invasion (Figure 3f).

Advanced‐stage GBC (T2) presented as a large, elevated lesion under WLI, with a papillary surface structure, although some areas were non‐structured (Figure 3g). NBI magnification revealed neovascularization and greater vascular disparity than T1 GBCs (Figure 3h). Histopathological examination revealed adenocarcinoma with small to medium‐sized tubular infiltration, accompanied by a fibrous stromal reaction extending into the subserosa (Figure 3i).

Advanced‐stage GBC (T3; with liver invasion) was observed under WLI as a large, partially submucosal, tumor‐like nodule (Figure 3j). The surface structure was amorphous. NBI magnification revealed irregular vessel images with neovascularization and destructive vessels (Figure 3k). Histopathological examination revealed a tubular adenocarcinoma with fused glandular tubular and pectinate structures, which had proliferated invasively into the liver parenchyma (Figure 3l). Based on these results, we outlined the endoscopically visualized features of each GB lesion (Table 1).

**TABLE 1:** Endoscopically visualized features of each type of gallbladder lesion.

Diagnosis	Surface structure (WLI)	Vascular structure (NBI magnification)
Normal GB	Regular, slightly elevated mucosa	Regular, no dilation
Adenomyomatosis of GB	Regular, highly elevated mucosa	Regular, no dilation
Cholecystolithiasis	Regular, slightly elevated mucosa	Regular, slightly dilated
Cholesterol polyp	Yellowish, spherical, elevated	Regular, no dilation
Hyperplastic polyp	Reddish, elevated	Regular, slightly dilated
Xanthogranulomatous cholecystitis	Non‐structure, scarring	Irregular, uniformly dilated
Early‐stage GBC	Papillary like a salmon roe, elevated	Irregular, caliber change
Advanced‐stage GBC	Papillary, non‐structure, elevated	Irregular, neovascularization, destructive vessels

Abbreviations: GB: gallbladder, GBC: gallbladder carcinoma, NBI: narrow‐band imaging, WLI: white light imaging.

### Interobserver agreement

Interobserver agreement (Table S2) was almost perfect for caliber change and loose vessel area (κ > 0.8). In addition, non‐uniformity had substantial agreement (κ = 0.6–0.8), while regular, absent, and dilation had moderate agreement (κ = 0.4–0.6).

### Comparison of benign elevated lesions and GBCs

Regarding surface structure, GBCs (Table 2) tended to have a more irregular surface (83.3% vs. 16.7%) than BELs. BELs showed no irregularities, except for XGC. T1 GBCs were regular, whereas the remaining GBCs showed irregular structures. Absent surface structures were found in advanced GBCs but not in benign elevated lesions (BELs) without XGC and T1 GBCs.

**TABLE 2:** Comparison of benign elevated lesion and gallbladder carcinoma.

	Benign elevated lesion *n* = 6	GB carcinoma *n* = 6	*p*
Surface structure			
Regular (±)	5/1	1/5	0.0801
Absent (±)	1/5	4/2	0.242
Vascular structure			
Dilation (±)	2/4	6/0	0.0606
Meandering (±)	1/5	6/0	0.0152
Caliber change (±)	0/6	6/0	0.00216
Non‐uniformity (±)	0/6	6/0	0.00216
Loose vessel areas (±)	0/6	4/2	0.182

Abbreviation: GB: gallbladder.

The microvascular findings showed significant differences between the two groups in meandering, caliber change, and non‐uniformity as follows: dilation (33.3% vs. 100%, *p* = 0.0606), meandering (16.7% vs. 100%, *p* = 0.0152), caliber change (0% vs. 100%, *p* = 0.00216), non‐uniformity (0% vs. 100%, *p* = 0.00216), and loose vessel areas and interruptions of thick vessels (0% vs. 66.7%, *p* = 0.182).

## DISCUSSION

To the best of our knowledge, this is the first study to evaluate the endoscopic characteristics of GB lesions under magnified NBI. By observing GB lesions endoscopically, the visualized findings shown in Table 1 were documented for each lesion type. These observations suggested the presence of characteristic differences between BELs and GBCs. In this study, we clarified the differences and trends in surface and vascular structures by comparing the NBI‐magnified endoscopic findings of BELs and GBCs, as presented in Table 2. Most GBCs, except for T1a cancer, exhibited irregular surface structures, whereas BELs, except for XGC, displayed a regular pattern. Concerning vascular structures, all GBCs exhibited meandering, caliber changes, and nonuniformity, features that were absent in BELs. Among BELs, XGC has irregular or absent surface structures, and although dilated vessels were observed, there were no caliber changes. When comparing XGC (Figure 2g,h) with GBC (Figure 3), the surface structural features appear somewhat similar. However, GBC exhibits irregular vascular distribution and significant caliber changes. Despite the limited sample size, a comparison between BELs and GBC revealed significant differences in vascular features, including meandering, caliber changes, and non‐uniformity (Table 2). Among these findings, high interobserver agreement was noted for caliber changes and non‐uniformity (Table S2), suggesting that these features may be useful endoscopic markers for differentiating BELs from GBC if similar findings are observed in a larger number of cases. XGC is a benign disease that is difficult to differentiate from GBC using any imaging modality, including computed tomography, magnetic resonance imaging, and endoscopic ultrasound.^17^ In clinical practice, some patients with XGC are misdiagnosed with GBC, leading to unnecessary radical surgery or incorrect diagnosis of advanced GBC. Should NBI magnification reveal distinct microvascular patterns that differentiate XGC from GBC, it would hold significant value in clinical practice.

In this study, when GBC cases were stratified by tumor invasion depth (T1–T3), different endoscopic findings were observed in both surface and vascular structures (Figure 3). These endoscopic characteristics of GBC could potentially vary according to the depth of invasion and disease progression. However, various GB adenocarcinomas have well‐differentiated papillotubular components at the tumor surface^18^ and show moderate‐to‐poor differentiation in the deeply invasive parts associated with desmoplastic stromal reactions. Our findings also showed that despite being T2 cancers, several areas retained papillary structures, with only a few regions exhibiting a loss of surface structure. Applying the diagnostic criteria established for gastric or colorectal cancers directly to GB lesions is challenging, and more cases will be necessary to determine endoscopic findings for GB lesions.

Few previous have observed the inside of GB using transpapillary peroral cholangioscopy. ^8^, ^9^, ^19^ The technical success rate with conventional peroral cholangioscopy is only 36.4%,^20^ owing to various reasons, including the thinness of the cystic duct and its spiral structure. Recently, a novel ultrafine peroral cholangioscopy capable of delineating GB lesions has been developed; the authors reported a technical success rate for insertion into the GB of 87.5%, with an adverse event rate of 6.3%.^19^ Currently, GB lesions are only observable under WLI; however, developing slim cholangioscopes that can perform IEE, such as NBI, is feasible. Magnified endoscopic observations of GB lesions, which we conducted using an explanted GB, have significant *in vivo* applications. Our study, characterizing the magnified endoscopic findings of GB lesions, will help to facilitate more accurate diagnosis.

The present study has some limitations. First, the study sample size was small, which might have resulted in selection bias and reduced external validity. In particular, flat‐type GBCs were not included, and observations of all types were not feasible. Future studies should include a large sample size and observe all types. Second, as the observations were conducted *ex vivo* immediately following cholecystectomy rather than in vivo within the GB, the findings may differ.

In conclusion, this study was the first to use WLI and NBI magnification to clarify the characteristics of each GB lesion. The endoscopic images of different GB lesions were characterized. This preliminary classification will contribute to innovative image diagnosis and targeted biopsy when diagnosing GB lesions under direct vision. Further studies remain warranted to confirm our findings.

## CONFLICT OF INTEREST STATEMENT

Hideki Kobara is an Associate Editor of DEN Open.

## ETHICS STATEMENT

This study was approved by the Institutional Ethics Review Board of the two participating facilities (Ethics Committee Approval Nos. – Kagawa Rosai Hospital: R2‐22, HITO Medical Center: 20210507002).

## PATIENT CONSENT STATEMENT

Written informed consent for this study was obtained from all patients before they underwent cholecystectomy.

## CLINICAL TRIAL REGISTRATION

N/A

## Supporting information



Table S1. Patient characteristics

Table S2. Interobserver agreement (*K* value)

Figure S1. Representative endoscopic images of vascular patterns in gallbladder lesions under narrow‐band imaging (NBI) magnification.
