# Supplementary material for: Endoscopic findings of gallbladder lesions evaluated with image‐enhanced endoscopy: A preliminary study using resected gallbladders
Source: DEN Open. 2025 May 3;6(1):e70136. doi: 10.1002/deo2.70136 (PMC12048906; doi:10.1002/deo2.70136)
Supplement: Supplementary file 1 — Table S1. Patient characteristics [file DEO2-6-e70136-s001.docx]

**Table S1. Patient characteristics**

| Patients, n | | | | | 50 |
| --- | --- | --- | --- | --- | --- |
| Sex, male/female, n | | | |  | 24/26 |
| Age (years), median [range] | | | |  | 67 [38-90] |
|  |  |  |  |  |  |
| Indication for cholecystectomy, n | | | |  |  |
|  | GB carcinoma | | |  | 6 |
|  | Cholecystolithiasis | | |  | 29 |
|  | GB polyp | | |  | 8 |
|  | Adenomyomatosis of the GB | | |  | 2 |
|  | Xanthogranulomatous cholecystitis | | |  | 1 |
|  | Gastric cancer | | |  | 4 |
|  |  |  |  |  |  |
| Pathological diagnosis after cholecystectomy, n | | | |  |  |
|  | No evidence of malignancy | | |  | 6 |
|  | Chronic cholecystitis, stone | | |  | 26 |
|  | Cholesterolosis, stone | | |  | 2 |
|  | Cholesterol polyp | | |  | 4 |
|  | Hyperplastic polyp | | |  | 1 |
|  | Adenomyomatosis of the GB | | |  | 4 |
|  | Xanthogranulomatous cholecystitis | | |  | 1 |
|  | Adenocarcinoma | | T1a |  | 1 |
|  |  |  | T1b |  | 1 |
|  |  |  | T2 |  | 2 |
|  |  |  | T3 |  | 2 |
|  |  |  |  |  |  |

Cancer diagnosis followed the 8^th^ edition of the “TNM Classification of Malignant Tumors” established by the Union for International Cancer Control (UICC).

GB: gallbladder.
