# Supplementary material for: Endoscopic findings of gallbladder lesions evaluated with image‐enhanced endoscopy: A preliminary study using resected gallbladders
Source: DEN Open. 2025 May 3;6(1):e70136. doi: 10.1002/deo2.70136 (PMC12048906; doi:10.1002/deo2.70136)
Supplement: Supplementary file 2 — Table S2. Interobserver agreement (K value) [file DEO2-6-e70136-s003.docx]

**Table S2. Interobserver agreement (κ value)**

|  | | Interobserver agreement |
| --- | --- | --- |
| Surface structure | |  |
|  | Regular | 0.462 |
|  | Absent | 0.588 |
| Vascular structure | |  |
|  | Dilation | 0.462 |
|  | Meandering | 0.364 |
|  | Caliber change | 1 |
|  | Non-uniformity | 0.72 |
|  | Loose vessel area | 1 |
