# Supplementary figures and images for: Endoscopic findings of gallbladder lesions evaluated with image‐enhanced endoscopy: A preliminary study using resected gallbladders
Source: DEN Open. 2025 May 3;6(1):e70136. doi: 10.1002/deo2.70136 (PMC12048906; doi:10.1002/deo2.70136)

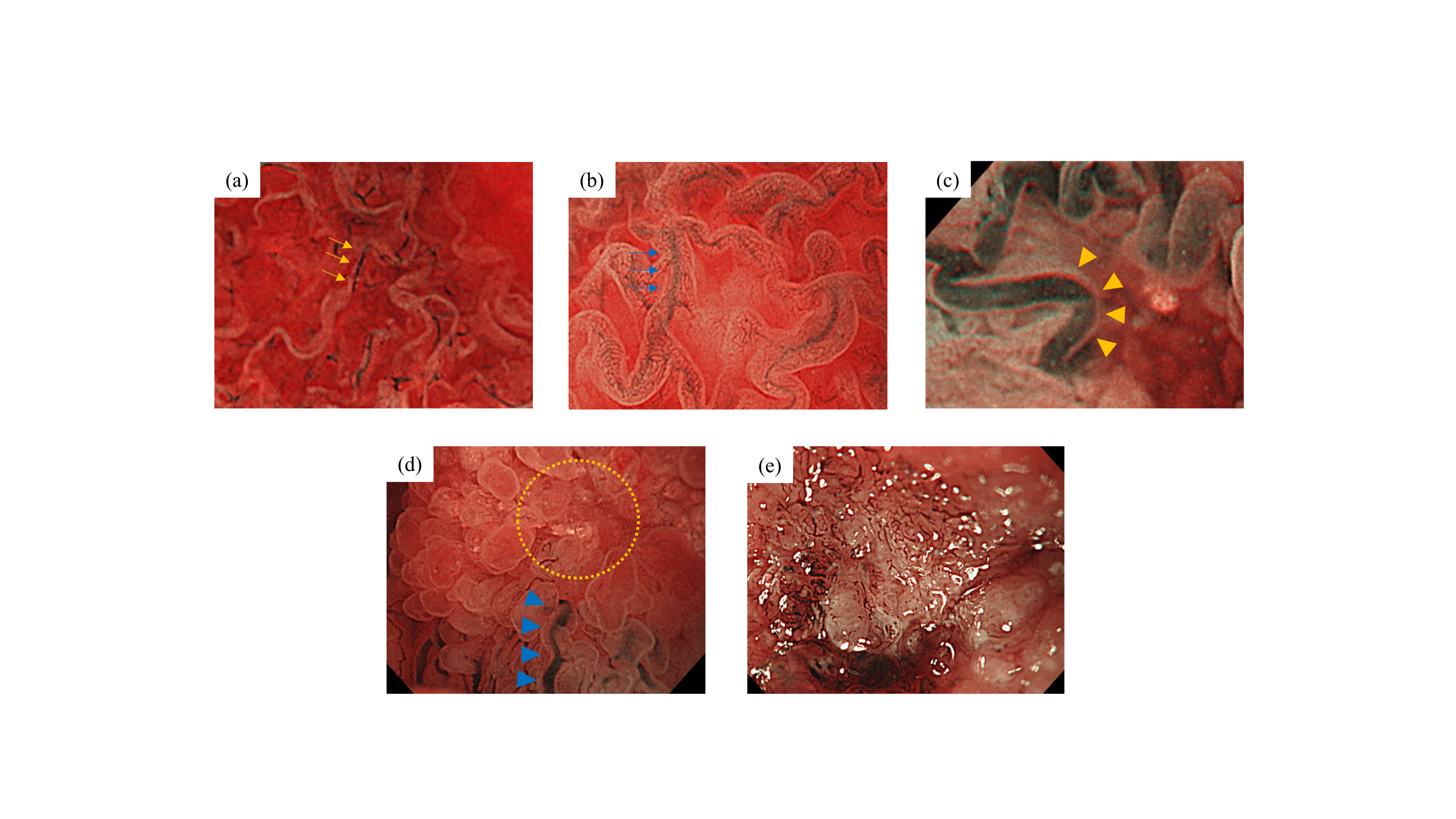

Supplement: Supplementary file 3 — Figure S1. Representative endoscopic images of vascular patterns in gallbladder lesions under narrow‐band imaging (NBI) magnification. [file DEO2-6-e70136-s002.tif]
